# BMI as a Mediator in the Relationship Between Dietary Trace Elements and Type 2 Diabetes Mellitus: Findings from a Rural Cohort

**DOI:** 10.3390/nu17172875

**Published:** 2025-09-05

**Authors:** Jianwei Wang, Biwen Shi, Haiyang Li, Yuqian Li, Zhenxing Mao, Chongjian Wang, Jian Hou, Yuan Tian, Linlin Li

**Affiliations:** 1 Department of Epidemiology and Health Statistics, College of Public Health, Zhengzhou University, Zhengzhou 450001, China; 15716690965@163.com (J.W.); 18336367622@163.com (B.S.); l15263964625@163.com (H.L.); maozhr@gmail.com (Z.M.); tjwcj2005@126.com (C.W.); 13667176505@163.com (J.H.); ty163email@163.com (Y.T.); 2 Department of Clinical Pharmacology, School of Pharmaceutical Science, Zhengzhou University, Zhengzhou 450001, China; liyuqian0214@126.com

**Keywords:** trace elements, type 2 diabetes mellitus, body mass index, cohort study

## Abstract

**Objectives**: This study aims to examine the relationship between dietary trace elements and Type 2 diabetes mellitus (T2DM), as well as to assess the influence of body mass index (BMI) on this relationship. **Methods**: A total of 38,384 participants participated in this study. Dietary intakes of iron, copper, zinc, heme iron, and non-heme iron were assessed using validated food frequency questionnaires. The odds ratio (OR) and 95% confidence interval (CI) were calculated using the logistic regression model to evaluate the association of dietary intake of iron, copper, zinc, heme iron, and non-heme iron with T2DM. Restrictive cubic splines (RCS) were used to explore the dose–response relationship. In addition, causal mediation analysis was used to explore the role of BMI. **Results**: After adjusting for the relevant covariates, the highest quartile (Q4) compared with the lowest quartile (Q1) the odds ratios and 95% confidence intervals of iron, heme iron, non-heme iron, copper, and zinc between T2DM were 0.81 (0.70–0.92), 0.81 (0.70–0.92), 0.79 (0.70–0.90), 0.64 (0.77–0.72), and 0.65 (0.55–0.78), respectively. The RCS results showed that the hazards of copper and heme iron in T2DM decreased with the increase in dose (*p*_-non_ < 0.05). The results of the mediation analysis showed that BMI mediated the association between dietary trace elements and T2DM. Furthermore, subgroup analysis showed the same results. **Conclusions**: This study indicates that moderate intake of dietary trace elements may help reduce the incidence of T2DM in rural areas. BMI can mediate the association between the two.

## 1. Introduction

In recent years, the global incidence of type 2 diabetes mellitus (T2DM) has risen steadily, making it become a major public health problem [1]. According to projections from the International Diabetes Federation (IDF), the global number of people with diabetes is anticipated to increase by 46% by 2045, significantly expanding the affected population [2,3]. In China, the number of diabetes patients is expected to rise to 130 million by 2030, with annual healthcare expenditures potentially hitting $490 billion [4,5,6].

The prevalence of T2DM is influenced by several factors, including genetics, environment, socioeconomic status and lifestyle choices, especially dietary factors [7,8,9]. Iron, copper, and zinc, as necessary trace elements, play key roles in regulating insulin sensitivity and maintaining blood glucose homeostasis. However, studies have shown that both excessive and insufficient intake of these elements can disrupt glucose regulation, thereby influencing the onset and progression of T2DM; for instance, excess iron accumulation can lead to oxidative stress and inflammation, which may impair insulin signaling pathways [10,11]. Research on dietary iron, copper, and zinc in relation to T2DM has yielded inconsistent results, with the most conflicting evidence emerging for heme iron and copper intake [12,13,14,15,16]. Moreover, most of these studies have focused on Western populations, and there are limited studies on the Chinese population, especially in rural areas of China. Given the differences in dietary habits, socioeconomic status, and ethnic backgrounds, the findings from Western populations may not be directly applicable to the Chinese population.

Body mass index (BMI) is a commonly used indicator for assessing the degree of obesity, and obesity has been widely regarded as an important risk factor for T2DM [17,18]. In addition, there is growing evidence that dietary nutrients can influence weight regulation and fat accumulation, thereby modulating diabetes risk by modifying obesity [19,20,21]. Given these biological mechanisms, the association between dietary trace element intake and the risk of type 2 diabetes may be partially mediated by BMI. Therefore, focusing on the rural population in China and evaluating the mediating relationship between minerals and BMI can help to deeply reveal the potential mechanism between dietary trace elements and T2DM.

Therefore, this study aims to investigate the relationship between dietary intakes of iron, copper, zinc, heme iron, non-heme iron, and the prevalence risk of T2DM in Chinese adults. Additionally, we analyze the potential mediating role of BMI in the relationship between these micronutrients and T2DM and examine their combined effects.

## 2. Materials and Methods

### 2.1. Study Participants

The Henan Rural Cohort was established in five rural areas of China’s Henan Province during 2015 to 2017, including 39,259 permanent residents. Details of the cohort have been previously published [22]. For our study, we excluded participants with missing dietary intake data (*n* = 79), unreasonable dietary intake data (*n* = 318), baseline diagnoses of type 1 diabetes (*n* = 67), renal failure (*n* = 19), or a history of tumors (*n* = 392), resulting in a final sample of 38,384 participants (Supplemental Figure S1). All participants provided written informed consent.

### 2.2. Assessment of Dietary Iron, Copper, and Zinc Intakes

The frequencies and quantities of each food or beverage consumed by the participants were collected using a food frequency questionnaire (FFQ). Based on the China Food Composition Table (6th Edition), we multiplied the frequency of each food based on its nutrient composition per 100 g, then summed all food items to calculate the average daily intake of iron, copper, and zinc. Additionally, 40% of the total iron content from animal tissues was considered heme iron intake [23]. Non-heme iron was calculated by the total iron minus the heme iron. All food and nutrient intakes were energy-adjusted using the residuals method [24]. The validity of the FFQ was evaluated by three consecutive 24 h ration evaluations, which showed good reproducibility and reasonable validity in dietary components [25].

### 2.3. Assessment of Covariates

Details on general demographic characteristics and lifestyle were obtained by trained researchers through face-to-face interviews and structured questionnaires. This information includes smoking, drinking, education level, marital status, and economic status. Physical activity levels were categorized as low, moderate, or vigorous based on the validated Chinese version of the International Physical Activity Questionnaire (IPAQ) [26]. BMI is calculated by dividing weight (kg) by the square of height (m).

### 2.4. Definition of T2DM

According to the American Diabetes Association (ADA) 2013 Diagnostic Criteria [27], T2DM was diagnosed if any of the following conditions were met: (1) fasting plasma glucose (FPG) ≥ 7.0 mmol/L, (2) self-reported diagnosis by a healthcare professional, or (3) use of insulin or other glucose-lowering medications within the last two weeks.

### 2.5. Statistical Analysis

Baseline characteristics of participants were grouped by whether they had diabetes or not. Continuous variables were reported as mean ± standard deviation (SD), and categorical variables were reported as frequencies (*n*) and percentages (%). The independent sample *t*-test or U test was used to assess differences in continuous variables, while the χ^2^ test was used for categorical variables. Multivariate logistic regression was applied to evaluate the relationships between dietary iron, copper, zinc, and T2DM. Model 1 was adjusted for age and gender. Model 2 was additionally adjusted for covariates such as education, smoking status, and alcohol consumption. Model 3 built on Model 2 with additional adjustments for total energy, carbohydrate, cereal fiber, cholesterol, and protein intakes. The dose–response relationships between dietary iron, copper, zinc, and T2DM risk was examined using restricted cubic splines (RCS). Additionally, we conducted mediation analysis using SPSS to explore whether BMI mediates the relationships between dietary iron, copper, zinc intake, and T2DM. Moreover, interaction terms were generated by multiplying the median intakes of iron and copper (mg/day) and the recommended zinc intake by stratified variables and were added to the model to explore joint effects.

All statistical analyses were performed using SPSS version 22.0 or R version 4.1.2. Two-tailed *p* values < 0.05 were considered statistically significant.

## 3. Results

### 3.1. Characteristics of the Study Participants

The participants’ baseline characteristics are summarized in Table 1. Of the participants, 15,147 were male and 23,237 were female, with a type 2 diabetes prevalence of 9.4%. Compared with the healthy participants, those with T2DM were more likely to be female, be older, have lower education and income levels, have a higher BMI, be less likely to smoke or drink, and be more likely to have hypertension and a diabetes family history. Additionally, in terms of diet, patients consumed less dietary iron, copper, zinc, heme iron, energy, and cholesterol but more grain fiber than the non-diabetic participants (all *p* values < 0.05).

### 3.2. Association Between Dietary Trace Elements and T2DM

Table 2 presents the odds ratios (OR) and 95% confidence intervals (CI) for dietary iron, copper, and zinc intake in relation to the prevalence risk of T2DM. After accounting for relevant factors, the multivariate OR (95% CI) for the highest quartile (Q4) versus the lowest quartile (Q1) was 0.81 (0.70–0.92) for iron, 0.69 (0.60–0.80) for heme iron, and 0.79 (0.70–0.90) for non-heme iron. In all three models, higher copper intake was consistently associated with a decreased prevalence risk of T2DM: Model 1 (OR: 0.80; 95% CI: 0.72–0.89), Model 2 (OR: 0.81; 95% CI: 0.73–0.90), and Model 3 (OR: 0.64; 95% CI: 0.57–0.72). In Model 3, higher zinc intake was linked to a decreased prevalence risk of developing T2DM (OR: 0.65; 95% CI: 0.55–0.78).

**Figure 1:**
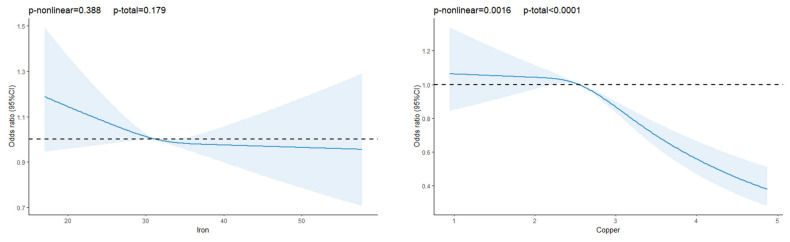

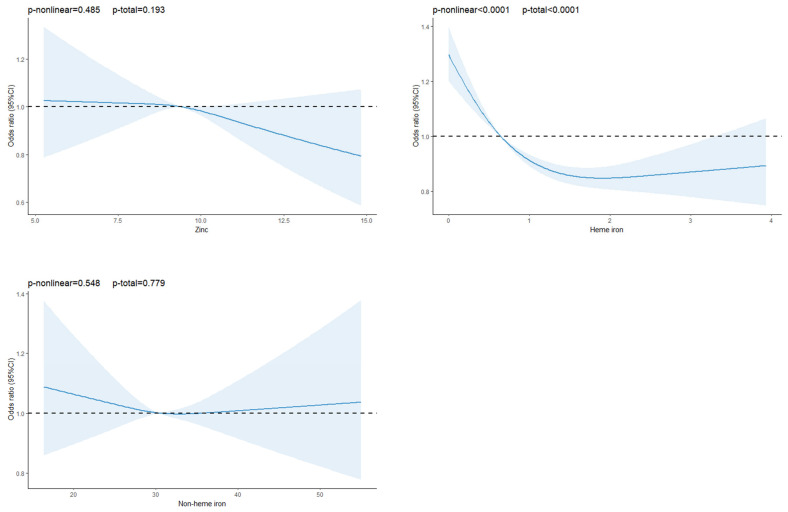
OR and 95% CIs for the risk of T2DM according to dietary intake of iron, copper, zinc, heme iron and non-heme iron, respectively, in model 3. CI, confidence interval; OR, odds ratio.

As shown in Figure 1, restricted cubic spline (RCS) analysis revealed inverse dose–response relationships between dietary intakes of iron, copper, zinc, and the prevalence risk of T2DM. Notably, the associations for copper and heme iron were nonlinear (*p*_-nonlinearity_ = 0.0016 and < 0.0001, respectively; *p*_-overall_ < 0.0001 for both). For copper, the risk of T2DM decreased with increasing intake, with the greatest prevalence risk reduction observed at intakes above 2.54 mg/day, beyond which the association leveled off. For heme iron, the inverse association with T2DM prevalence risk became evident when intake exceeded 0.65 mg/day; however, the protective effect was gradually diminished at higher intake levels.

### 3.3. Stratification Analysis

The subgroup analysis results showed that in most subgroups, the link between iron intake and the prevalence risk of T2DM remains significant (Supplemental Figure S2). Copper intake was similarly associated with reduced T2DM prevalence risk in most subgroups, excluding former or current smokers and drinkers. However, for zinc and heme iron, no significant associations were found in participants under 55 years of age, those with family histories of diabetes for zinc, participants with a BMI < 24, former or current smokers or drinkers, or those with a family history of diabetes for heme iron (Supplemental Figure S3). Non-heme iron intake was significantly associated with T2DM prevalence risk among women, participants aged ≥ 55, non-drinkers, those with a per capita monthly income < 1000 yuan, low physical activity levels, and those without family histories of diabetes.

### 3.4. Mediation Effects of BMI on Dietary Trace Elements and T2DM Association

**Figure 2:**
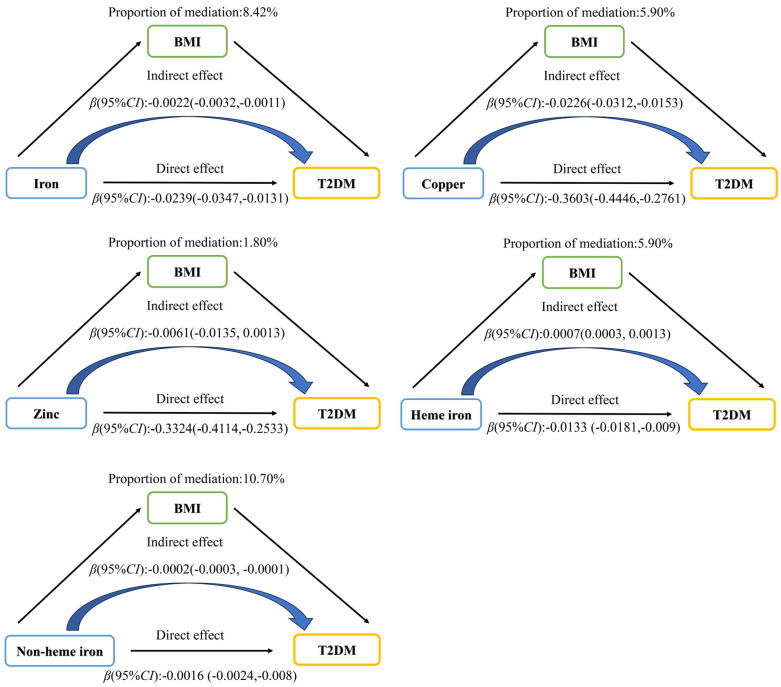
The mediating effect of BMI on the association between dietary iron, copper, zinc, heme iron, non-heme iron intake, and T2DM.

Mediation analysis showed that BMI played a partial mediating role in the associations between dietary iron, copper, zinc intake and the risk of T2DM (Figure 2). The direct effect of dietary iron on T2DM was −0.0239 (95% CI: −0.037, −0.0131, *p* < 0.001), with BMI accounting for 8.42% of the mediation. For copper, zinc, and non-heme iron, BMI mediated 5.90%, 1.80%, and 10.70% of the associations, respectively. As for heme iron, its indirect effect was 0.0007 (0.0003, 0.0013), and its direct effect was −0.0133 (−0.0181, −0.009).

### 3.5. Interaction Analysis and Combined Effects

In the interaction analysis, we observed that the correlation between dietary copper intake and the prevalence risk of T2DM was significantly different by gender (*p*_-interaction_ < 0.001), age (*p*_-interaction_ < 0.002), smoking status (*p*_-interaction_ < 0.001), and alcohol consumption (*p*_-interaction_ < 0.005). Specifically, among women, the elderly, non-smokers, and non-drinkers, the negative correlation between copper intake and the prevalence risk of T2DM was stronger. For zinc, a significant interaction was observed with gender (*p_-_*_interaction_ = 0.001), with a more pronounced protective effect in women as compared with men. Similarly, for heme iron, significant interactions were found with gender (*p*_-interaction_ < 0.001), smoking (*p*_-interaction_ = 0.001), drinking (*p*_-interaction_ = 0.008), and physical activity (*p*_-interaction_ = 0.011). The protective association of heme iron was stronger in women, non-smokers, non-drinkers, and individuals with higher physical activity levels. No significant interaction effects were observed in other stratified analyses.

The combined effects of dietary intake of iron–zinc, copper–zinc, and iron–copper on the risk of T2DM are presented in Supplemental Tables S1 and S2. After adjusting for potential factors, the highest dietary intakes of both iron and zinc were associated with a reduced prevalence risk of T2DM, with an odds ratio (OR) of 0.90 (95% CI: 0.66–1.23) compared with the reference group. For participants with the highest intakes of both copper and zinc, the prevalence risk of T2DM was further reduced (OR: 0.85, 95% CI: 0.62–1.16). In the group with the highest intakes of both iron and copper, the prevalence risk of T2DM was even lower (OR: 0.75, 95% CI: 0.67–0.84).

## 4. Discussion

In this large study of rural adults in China, we found a significant inverse association between dietary intakes of iron, copper, and zinc and the prevalence risk of T2DM. The protective effects of dietary iron, copper, and zinc on T2DM were not evident in people who smoked, drank alcohol, and had family histories of diabetes.

Our findings on dietary iron are supported by a study that used UK Biobank data, which showed a strong protective effect of iron against T2DM [28]. In contrast, another study suggested a positive association between higher dietary iron and non-heme iron intake and the risk of T2DM [12]. However, a meta-analysis of 11 longitudinal studies, encompassing 323,788 participants and 28,837 cases of T2DM, found no significant association between dietary iron or non-heme iron intake and T2DM risk [29]. The reasons for these differences in results are unclear and may be influenced by factors such as the form of iron. On the other hand, contrary to our results on heme iron, many cross-sectional studies have consistently shown that a higher heme iron intake is significantly associated with an increased risk of T2DM [12,15,16,30]. The divergence in these findings may be partly explained by differences in average heme iron intake. Additionally, regional differences and dietary patterns could contribute to the inconsistencies. Given the complexity and uncertainty of these findings, more high-quality prospective studies are needed to explore their associations.

Research on dietary copper intake remained relatively scarce. A prospective study in Japan found a positive association between higher copper intake and an increased risk of T2DM [12]. However, in our study, the restricted cubic spline plot revealed that higher dietary copper intake was associated with a significantly reduced prevalence of T2DM. One possible explanation for the inconsistent results is the presence of potential confounding factors (intake of other minerals and vitamins). A prospective study that explored the relationship between dietary selenium, copper, and T2DM found that dietary selenium intake significantly modified the association between copper intake and T2DM risk [31]. Regarding the interaction between copper intake and smoking or drinking status, our analysis suggests that smoking and drinking increase the production of free radicals in the body. Excessive alcohol consumption, on the other hand, may impair liver function—a key organ in copper regulation—and long-term heavy drinking could disrupt normal copper metabolism. Therefore, when developing health recommendations to prevent T2DM, particular emphasis should be placed on the importance of limiting alcohol consumption or avoiding alcohol.

Our findings regarding dietary zinc were in agreement with several earlier studies [32,33,34,35]. However, an American study observed that dietary zinc intake is linked to an increased risk of metabolic syndrome, which is in contrast to our findings [36]. This discrepancy may be explained by differences in the sources of dietary zinc. The American study derived all its dietary zinc from red meat, whereas our study, along with others, included a variety of food sources not limited to red meat. Notably, several studies have indicated that a high intake of red meat is linked to an elevated risk of chronic diseases, including diabetes, cardiovascular disease, and coronary heart disease [37,38,39,40].

The association between these dietary trace elements and the risk of T2DM may be achieved through a variety of complex biological mechanisms. First, iron mainly participates in the transportation and storage of oxygen in the body and acts as a cofactor for various enzymes. It maintains blood glucose homeostasis by promoting insulin secretion. However, iron overload can catalyze the generation of reactive oxygen species (ROS) through the Fenton reaction, leading to oxidative stress, damaging pancreatic beta cells, and promoting insulin resistance [11,12]. The protective effect of copper may stem from its role as an essential component of ceruloplasmin, a protein with ferrous oxidase activity that promotes the transport and release of iron ions within cells, thereby preventing the Fenton reaction and the massive generation of hydroxyl radicals caused by the accumulation of free iron and reducing the risk of T2DM by alleviating oxidative stress [41,42]. Zinc is an important component of superoxide dismutase (SOD). It maintains the activity of enzymes by stabilizing their three-dimensional structure and plays an important antioxidant role. Oxidative stress is a key factor in triggering and maintaining chronic inflammatory responses. Chronic low-grade inflammation plays a core role in the pathogenesis of T2DM. The protective effect of zinc may stem from its indirect inhibition of inflammatory responses through antioxidant pathways, thereby reducing the risk of T2DM [32,33,34,35]. In the mediating effect analysis of BMI, we found that BMI showed a significant negative mediating effect in the relationships between dietary iron, copper, zinc, and non-heme iron and T2DM, while it showed a positive mediating effect in heme iron. This result indicates that these dietary trace elements may change an individual’s BMI level through some underlying mechanism, thereby indirectly altering the risk of T2DM. The mechanism may be related to the regulatory effect of trace elements on the composition of the intestinal flora and its metabolic function. Previous studies have shown that adding copper to a diet can change the relative abundance of intestinal microbiota such as firmicutes and warthobiota at the phylum level, promote the expression of liver fat-related genes, and increase liver lipid accumulation [43].

In addition, our study found that the combined effects of dietary iron, copper, and zinc on T2DM risk were smaller than their individual effects. The analysis of the interactions between iron and copper, iron and zinc, and copper and zinc revealed positive additive interactions for all pairwise combinations. The precise mechanisms behind the associations remain unclear. However, they may be related to the competitive inhibition of their absorptions in the small intestine [14,44]. Studies have shown that excessive iron intake can reduce copper- and zinc-dependent superoxide dismutase (CuZn-SOD) activities, potentially impairing copper absorption. Infant formulas with high iron content, for example, have been shown to decrease copper absorption and status compared with formulas with lower iron contents [13,45]. These results suggest that dietary planning aimed at preventing T2DM should carefully consider the balance of iron, copper, and zinc intakes to minimize the risk of competitive inhibition, which could potentially reduce their beneficial effects on glucose metabolism.

This study has some limitations. First, as a cross-sectional study, it cannot establish a causal relationship between dietary intakes of iron, copper, zinc, and T2DM. Second, there are confounding factors that cannot be adjusted in response, such as sleep quality and other dietary factors. Third, there may be reverse causality. Diabetic patients may change their diets after diagnosis (due to medical advice or habit changes), which may affect the observed association. Fourth, the self-reporting nature of dietary and lifestyle data may introduce recall bias or social expectation bias. Fifth, we cannot include all dietary trace elements in the data, and there is a deficiency of certain trace nutrients. Finally, as this study is a cross-sectional study, it is not yet possible to determine whether it is dietary trace elements or the overall healthy dietary pattern that influences T2DM. In subsequent research, efforts should be made to strengthen the investigation into this association.

## 5. Conclusions

This study indicates that in rural China, moderate intakes of iron, copper, zinc, heme iron, and non-heme iron may reduce the prevalence risk of T2DM. Dietary plans for T2DM should emphasize balancing the intakes of iron, copper, and zinc to minimize potential mutual inhibition.

## Figures and Tables

**Table 1:** Participants’ characteristics according to type 2 diabetes status mellitus.

Characteristics	Healthy Participants (*N* = 34,768)	Diabetic Subjects (*N* = 3616)	Total (*N* = 38,384)	*p*
	Mean ± SD or *n* (%)	Mean ± SD or *n* (%)	Mean ± SD or *n* (%)	
Gender (%)				0.047
Male	13,776 (39.6)	1371 (37.9)	15,147 (39.5)	
Female	20,992 (60.4)	2245 (62.1)	23,237 (60.5)	
Culture (%)				<0.01
Primary school or below	15,189 (43.7)	2000 (55.3)	17,189 (44.8)	
Junior high school	14,107 (40.6)	1196 (33.1)	15,303 (39.9)	
Senior high school or below	5472 (15.7)	420 (11.6)	5892 (15.3)	
Marital status (%)				0.009
Married/cohabiting	31,271 (89.9)	3202 (88.6)	34,473 (89.8)	
Single/divorced/widowed	3497 (10.1)	414 (11.4)	3911 (10.2)	
Per capita monthly income (%)				<0.01
<500 RMB	12,234 (35.2)	1424 (39.4)	13,658 (35.6)	
500–1000 RMB	11,494 (33.0)	1160 (32.1)	12,654 (33.0)	
>1000 RMB	11,040 (31.8)	1032 (28.5)	12,072 (31.4)	
Exercise (%)				<0.01
Low	10,946 (31.5)	1411 (39.1)	12,357 (32.2)	
Moderate	13,235 (38.0)	1285 (35.5)	14,520 (37.8)	
High	10,587 (30.5)	920 (25.4)	11,507 (30.0)	
Smoking (%)				<0.01
Never	25,218 (72.5)	2730 (75.5)	27,948 (72.8)	
Former or current smoking	9550 (27.5)	886 (24.5)	10,436 (27.2)	
Drinking (%)				0.009
Never	26,809 (77.1)	2858 (79.0)	29,667 (77.3)	
Former or current drinking	7959 (22.9)	758 (21.0)	8717 (22.7)	
Hypertension (%)				<0.01
No	24,063 (69.2)	1765 (48.8)	25,828 (67.3)	
Yes	10,674 (30.7)	1849 (51.2)	12,523 (32.6)	
Family history of T2DM (%)				<0.01
No	33,530 (96.4)	3259 (90.1)	36,789 (95.8)	
Yes	1238 (3.6)	357 (9.9)	1595 (4.2)	
Age (years)	55.06 ± 12.35	60.36 ± 9.27	55.56 ± 12.19	<0.01
Iron (mg/day)	32.05 ± 5.02	31.91 ± 5.05	32.04 ± 5.02	0.104
Copper (mg/day)	2.62 ± 0.53	2.55 ± 0.49	2.61 ± 0.53	<0.01
Zinc (mg/day)	9.50 ± 1.14	9.46 ± 1.13	9.49 ± 1.14	0.090
Heme iron (mg/day)	0.99 ± 0.96	0.88 ± 0.92	0.98 ± 0.95	<0.01
Non-heme iron (mg/day)	31.06 ± 4.79	31.03 ± 4.84	31.06 ± 4.79	0.713
BMI (kg/m^2^)	24.70 ± 3.52	26.19 ± 3.70	24.84 ± 3.57	<0.01
Total energy (kcal/day)	1802.80 ± 540.56	1744.63 ± 547.89	1797.32 ± 541.51	<0.01
Carbohydrate (g/day)	317.58 ± 30.76	317.43 ± 30.13	317.56 ± 30.70	0.783
Cereal fiber (g/day)	6.42 ± 2.02	6.57 ± 2.11	6.43 ± 2.03	<0.01
Cholesterol (mg/day)	436.25 ± 289.77	422.98 ± 295.04	435.00 ± 290.29	0.009
Protein (g/day)	69.49 ± 7.77	69.54 ± 3.70	69.50 ± 7.77	0.727

RMB, renminbi; BMI, body mass index; T2DM, type 2 diabetes mellitus.

**Table 2:** Odds ratio (95% CI) for T2DM risk by quartile of iron, copper, zinc, heme iron, and non-heme iron.

Iron	Q1	Q2	Q3	Q4	*p*-Trend
Model 1	1.00 (ref)	1.01 (0.92–1.11)	0.96 (0.87–1.06)	0.99 (0.90–1.09)	0.694
Model 2	1.00 (ref)	1.03 (0.93–1.14)	1.02 (0.92–1.13)	1.09 (0.99–1.21)	0.111
Model 3	1.00 (ref)	0.91 (0.82–1.01)	0.85 (0.76–0.95)	0.81 (0.70–0.92)	0.002
Copper					
Model 1	1.00 (ref)	0.98 (0.89–1.08)	1.00 (0.90–1.10)	0.80 (0.72–0.89)	<0.01
Model 2	1.00 (ref)	0.97 (0.88–1.07)	1.00 (0.91–1.10)	0.81 (0.73–0.90)	<0.01
Model 3	1.00 (ref)	0.85 (0.77–0.94)	0.83 (0.75–0.93)	0.64 (0.57–0.72)	<0.01
Zinc					
Model 1	1.00 (ref)	1.05 (0.95–1.16)	1.08 (0.98–1.19)	1.11 (1.01–1.23)	0.036
Model 2	1.00 (ref)	1.07 (0.97–1.18)	1.10 (1.00–1.22)	1.15 (1.04–1.27)	0.008
Model 3	1.00 (ref)	0.89 (0.80–1.00)	0.81 (0.71–0.92)	0.65 (0.55–0.78)	<0.01
Heme iron					
Model 1	1.00 (ref)	0.92 (0.83–1.01)	0.97 (0.88–1.07)	0.94 (0.85–1.04)	0.499
Model 2	1.00 (ref)	0.94 (0.86–1.04)	1.01 (0.91–1.11)	0.99 (0.89–1.10)	0.760
Model 3	1.00 (ref)	0.90 (0.82–1.00)	0.88 (0.79–0.98)	0.69 (0.60–0.80)	<0.01
Non-heme iron					
Model 1	1.00 (ref)	0.98 (0.89–1.08)	0.95 (0.86–1.05)	0.99 (0.90–1.09)	0.764
Model 2	1.00 (ref)	1.01 (0.91–1.11)	1.01 (0.92–1.12)	1.08 (0.98–1.20)	0.114
Model 3	1.00 (ref)	0.89 (0.80–0.99)	0.84 (0.75–0.94)	0.79 (0.70–0.90)	0.001

Model 1: adjusted for age and sex. Model 2: Model 1 + education level, marital status, per capita monthly income, tobacco smoking, alcohol drinking, physical activity, hypertension, family history of diabetes, and BMI. Model 3: Model 2 + total energy intake and carbohydrates, ratio of MUFA-to-SFA, ratio of PUFA-to-SFA, cereal fiber, cholesterol, and protein. In addition, non-heme iron was adjusted to heme iron and heme iron was adjusted to non-heme iron.

## Data Availability

The data analyzed during the current study are available from the corresponding author upon reasonable request.

## Supplementary Materials

The following supporting information can be downloaded at https://www.mdpi.com/article/10.3390/nu17172875/s1, Figure S1: Flow chart of participant selection; Figure S2: Subgroup analysis of iron, copper, zinc on the risk of type 2 diabetes mellitus; Figure S3: Subgroup analysis of heme iron, non-heme iron on the risk of type 2 diabetes; Table S1: Relationship between dietary iron-zinc, copper-zinc intake and T2DM; Table S2: Association of dietary iron and copper intake with T2DM.
