# Supplementary material for: BMI as a Mediator in the Relationship Between Dietary Trace Elements and Type 2 Diabetes Mellitus: Findings from a Rural Cohort
Source: Nutrients. 2025 Sep 5;17(17):2875. doi: 10.3390/nu17172875 (PMC12430077; doi:10.3390/nu17172875)
Supplement: Supplementary file 1 [file nutrients-17-02875-s001.zip › nutrients-3819374-supplementary.pdf]

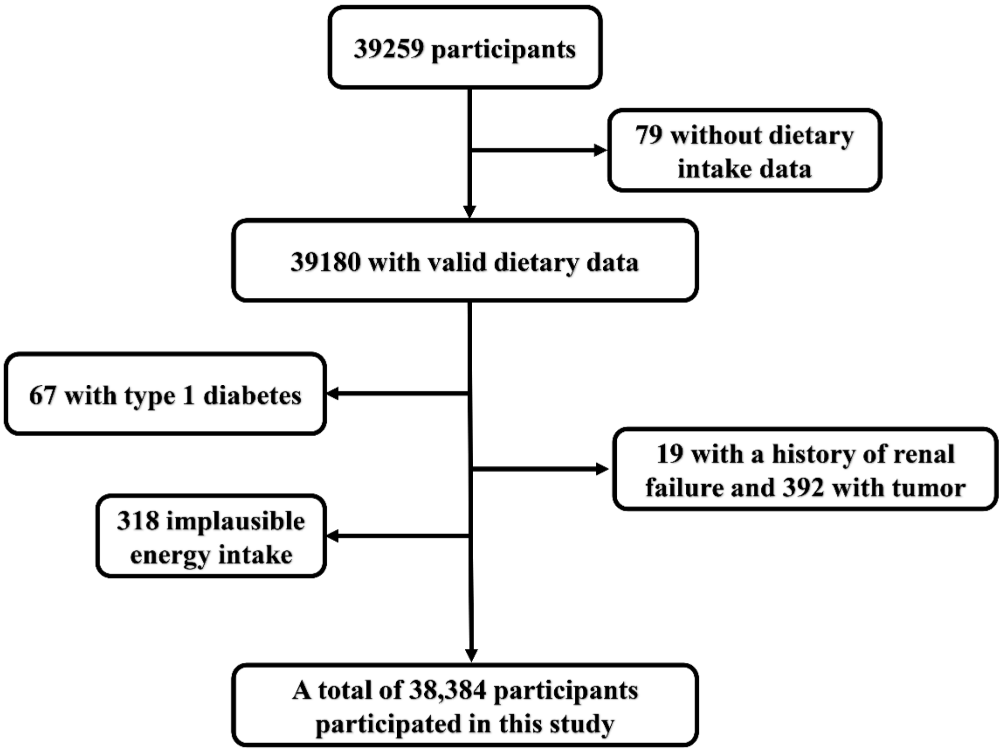

Figure S1 Flow chart of participant selection

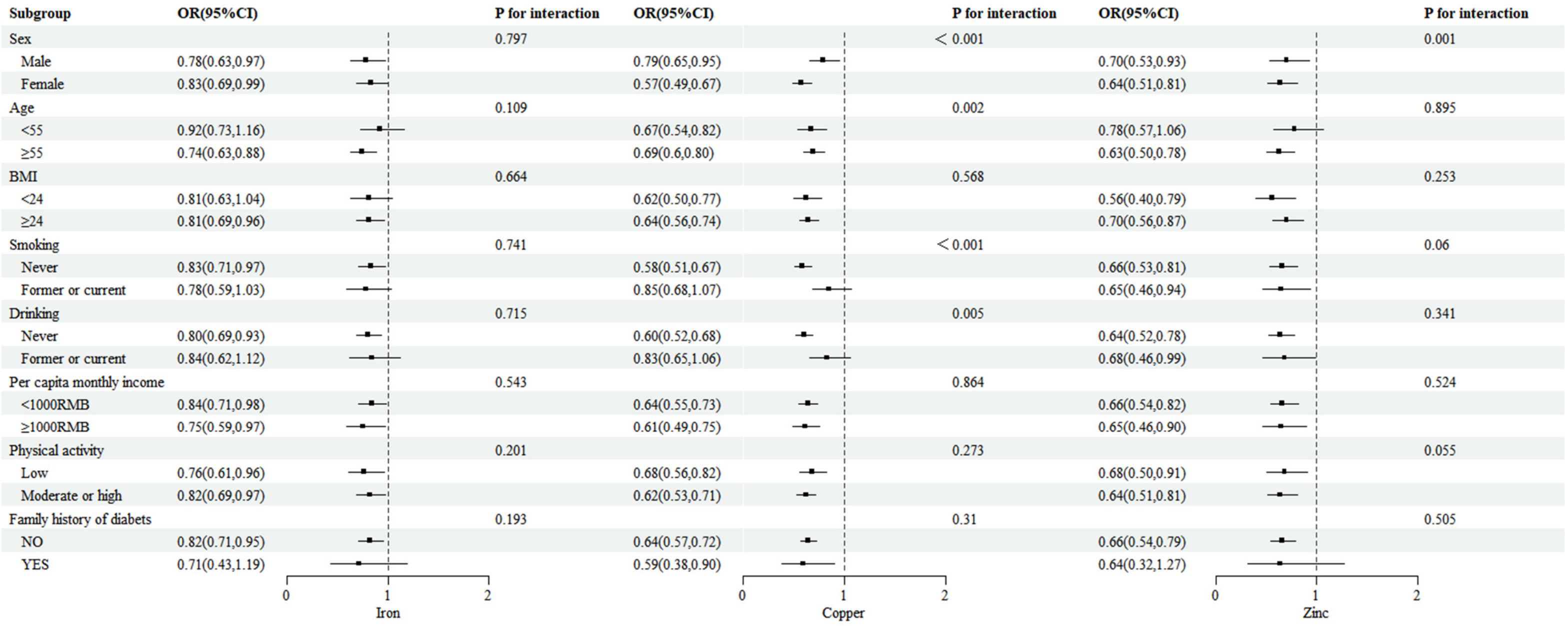

Figure S2 Subgroup analysis of iron, copper, zinc on the risk of type 2 diabetes mellitus. OR odds ratio, CI confidence interval, BMI body mass index

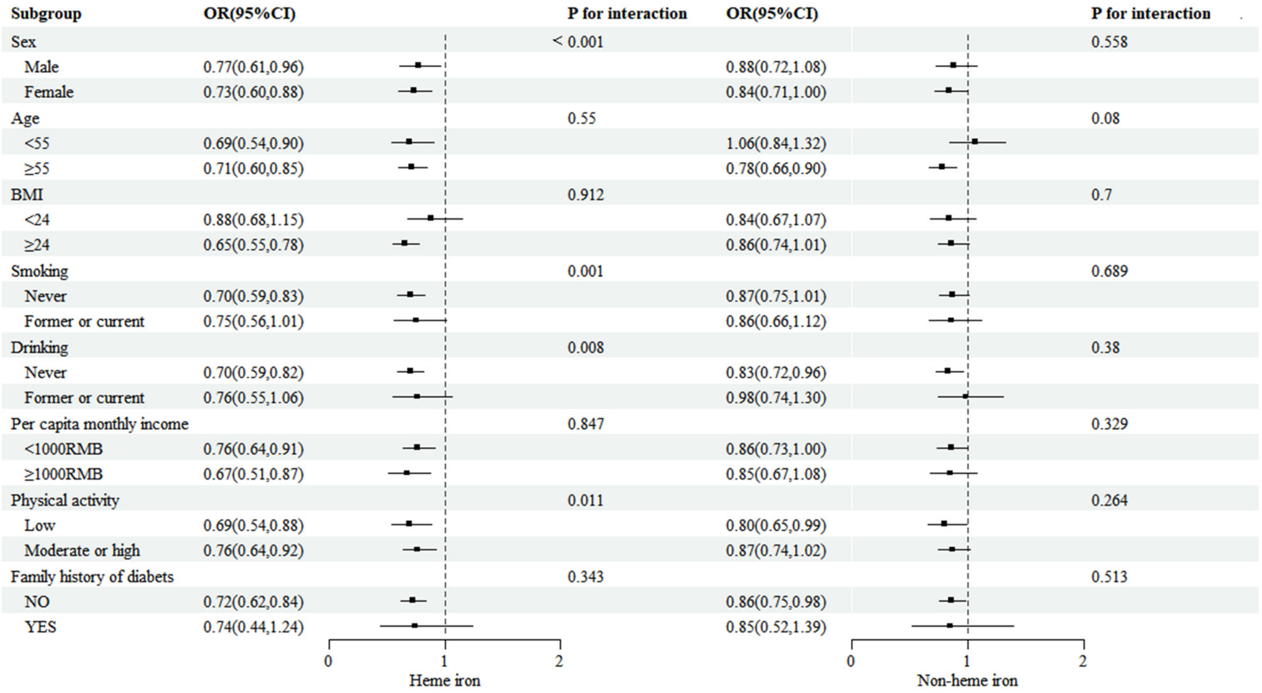

Figure S3 Subgroup analysis of heme iron, non-heme iron on the risk of type 2 diabetes. OR odds ratio, CI confidence interval, BMI body mass index

Variables		Zinc intake		AP	S
		< RDA	⩾RDA		
Model 3 Iron intake	< Median intake	1.00 (ref)	1.02(0.75-1.38)	-0.01	1.11
	⩾Median intake	0.89(0.79-1.01)	0.90(0.66-1.23)		
Model 3 copper intake	< Median intake	1.00 (ref)	1.09(0.80-1.49)	-0.15	7.50
	⩾Median intake	0.89(0.79-1.00)	0.85(0.62-1.16)		

Table S1 Relationship between dietary iron-zinc, copper - zinc intake and T2DM; RDA, recommended dietary allowance; AP, attributable proportion due to interaction; S; synergy index

Variables		Iron intake		AP	S
		< Median intake	⩾Median intake		
Model 3 copper intake	< Median intake	1.00 (ref)	0.95(0.85-1.06)	-0.08	1.32
	⩾Median intake	0.86(0.76-0.96)	0.75(0.67-0.84)		

Table S2 Association of dietary iron and copper intake with T2DM; RDA, recommended dietary allowance; AP, attributable proportion due to interaction; S; synergy index
